# New Books

**Published:** 2008-03

Apollo's Fire: Igniting America's Clean Energy Economy

Jay Inslee, Bracken Hendricks

Washington, DC:Island Press, 2007. 416 pp. ISBN: 1-59726-175-0, $25.95

Assessing Climate Change: Temperatures, Solar Radiation and Heat Balance

Donald Rapp

New York:Springer, 2008. 410 pp. ISBN: 978-3-540-76586-8, $179

Biological, Chemical, and Radiological Terrorism: Emergency Preparedness and Response for the Primary Care Physician

Alan Melnick

New York:Springer, 2008. 248 pp. ISBN: 978-0-387-47231-7, $39.95

Combined Exposures to Hydrogen Cyanide and Carbon Monoxide in Army Operations: Initial Report

Committee on Combined Exposures to Hydrogen Cyanide and Carbon Monoxide in Army Operations, Committee on Toxicology, National Research Council

Washington, DC:National Academies Press, 2008. 42 pp. ISBN: 978-0-309-11366-3, $18

Decontamination of Warfare Agents: Enzymatic Methods for the Removal of B/C Weapons

Andre Richardt, Marc-Michael Blum, eds.

Hoboken, NJ:John Wiley & Sons, Inc., 2008. 311 pp. ISBN: 978-3-527-31756-1, $160

Emergence: Contemporary Readings in Philosophy and Science

Mark A. Bedau, Paul Humphreys, eds.

Cambridge, MA:MIT Press, 2008. 482 pp. ISBN: 978-0-262-02621-5, $80

Encyclopedia of Genetics, Genomics, Proteomics, and Informatics

George Rédei

New York:Springer, 2008. 2,400 pp. ISBN: 978-1-4020-6755-6, $999

Environmental Justice in Latin America

David V. Carruthers, ed.

Cambridge, MA:MIT Press, 2008. 336 pp. ISBN: 978-0-262-03372-5, $62

Environmental Law, Policy, and Economics: Reclaiming the Environmental Agenda

Nicholas A. Ashford, Charles C. Caldart

Cambridge, MA:MIT Press, 2008. 1,088 pp. ISBN: 978-0-262-01238-6, $90

Essentials of Ecology, 3rd ed.

Colin R. Townsend, Michael Begon, John L. Harper

Hoboken, NJ:John Wiley & Sons, Inc., 2008. 532 pp. ISBN: 978-1-4051-5658-5, $84.95

Global Environmental Diplomacy: Negoatiating Environmental Agreements for the World, 1973–1992

Mostafa K. Tolba

Cambridge, MA:MIT Press, 2008. 232 pp. ISBN: 978-0-262-70122-8, $23

Groundwater Science and Policy: An International Overview

Phillippe Quevauviller, ed.

New York:Springer, 2008. 648 pp. ISBN: 978-0-85404-294-4, $199

Molecular Mechanisms of Cancer

Georg F. Weber

New York:Springer, 2007. 650 pp. ISBN: 978-1-4020-6015-1, $120

Pollution of Lakes and Rivers

John P. Smol

Hoboken, NJ:John Wiley & Sons, Inc., 2008. 396 pp. ISBN: 978-1-4051-5913-5, $59.95

Protecting the Gulf’s Marine Ecosystems from Pollution

A.H. Abuzinada, H.-J. Barth, F. Krupp, B. Böer, T.Z. Al Abdessalaam, eds.

New York:Springer, 2008. 285 pp. ISBN: 978-3-7643-7946-9, $99

Review of Toxicologic and Radiologic Risks to Military Personnel from Exposure to Depleted Uranium During and After Combat

Committee on Toxicologic and Radiologic Effects from Exposure to Depleted Uranium During and After Combat, Committee on Toxicology, National Research Council

Washington, DC:National Academies Press, 2008. 158 pp. ISBN: 978-0-309-11036-5, $37.50

The Green Building Revolution

Jerry Yudelson

Washington, DC:Island Press, 2007. 272 pp. ISBN: 1-59726-179-3, $25

The Illusion of Certainty: Health Benefits and Risks

Erik Rifkin, Edward Bouwer

New York:Springer, 2007. 244 pp. ISBN: 978-0-387-75165-8, $29.95

The Joy of Science

Richard A. Lockshin

New York:Springer, 2008. 450 pp. ISBN: 978-1-4020-6098-4, $89.95

The Option of Urbanism: Investing in a New American Dream

Christopher B. Leinberger

Washington, DC:Island Press, 2007. 224 pp. ISBN: 1-59726-136-X, $25.95

The Role of Theory in Advancing 21st Century Biology: Catalyzing Transformative Research

Committee on Defining and Advancing the Conceptual Basis of Biological Sciences in the 21st Century, National Research Council

Washington, DC:National Academies Press, 2008. 208 pp. ISBN: 978-0-309-11249-9, $45

The Toxicology and Biochemistry of Insecticides

Simon J. Yu

Boca Raton, FL:CRC Press, 2008. 296 pp. ISBN: 9781420059755, $99.95

Toxicity Testing in the 21st Century: A Vision and a Strategy

Committee on Toxicity Testing and Assessment of Environmental Agents, National Research Council

Washington, DC:National Academies Press, 2007. 216 pp. ISBN: 978-0-309-10992-5, $32.40

